# Factors that influence family and parental preferences and decision making for unscheduled paediatric healthcare: a systematic review protocol

**DOI:** 10.12688/hrbopenres.12897.2

**Published:** 2019-09-30

**Authors:** Emma Nicholson, Therese McDonnell, Moayed Hamza, Michael Barrett, Christopher Brunsdon, Gerard Bury, Martin Charlton, Claire Collins, Conor Deasy, Aoife De Brun, John Fitzsimons, Marie Galligan, Conor Hensey, Fiona Kiernan, Eilish McAuliffe

**Affiliations:** 1 UCD School of Nursing, Midwifery & Health Systems, University College Dublin, Dublin, Ireland; 2 Chilren's Health Ireland at Crumlin, Dublin 12, Ireland; 3 UCD School of Medicine, University College Dublin, Belfield, Dublin 4, Ireland; 4 National Children's Research Centre, Crumlin, Dublin 12, Ireland; 5 National Centre for Geocomputation, National University of Ireland, Maynooth, Maynooth, Co Kildare, Ireland; 6 Irish College of General Practitioners, Dublin 2, Ireland; 7 Cork University Hospital, Cork, Ireland; 8 Children's Health Ireland at Temple Street, Dublin 1, Ireland; 9 UCD Geary Institute, University College Dublin, Belfield, Dublin 4, Ireland

**Keywords:** paediatric healthcare, general practice, out of hours, emergency department, decision-making

## Abstract

There is a plethora of factors that dictate where parents and families choose to seek unscheduled healthcare for their child; and the complexity of these decisions can present a challenge for policy makers and healthcare planners as these behaviours can have a significant impact on resources in the health system. The systematic review will seek to identify the factors that influence parents’ and families’ preferences and decision making when seeking unscheduled paediatric healthcare.  Five databases will be searched for published studies (CINAHL, PubMed, SCOPUS, PsycInfo, EconLit) and grey literature will also be searched. Inclusion and exclusion criteria will be applied and articles assessed for quality. A narrative approach will be used to synthesise the evidence that emerges from the review. By collating the factors that influence decision-making and attendance at these services, the review can inform future health policies and strategies seeking to expand primary care to support the provision of accessible and responsive care. The systematic review will also inform the design of a discrete choice experiment (DCE) which will seek to determine parental and family preferences for unscheduled paediatric healthcare. Policies that seek to expand primary care and reduce hospital admissions from emergency departments need to be cognisant of the nuanced and complex factors that govern patients’ behaviour.

## Introduction

### Paediatric use of unscheduled healthcare

Paediatric populations account for a significant number of attendances at emergency departments (EDs) and general practices (GP) in Ireland ( Wren *et al*., 2017), with children aged under 6 alone constituting approximately 14% of overall visits to the ED ( Wren *et al*., 2017). Attendance rates at GPs for all age groups in Ireland are gradually increasing ( O’Callaghan *et al*., 2018) while EDs are also under increasing pressure due to rising presentations (Health Service Executive, 2017). While factors related to the health system and structure can influence visitation rates to the ED, patient factors also dictate where healthcare is sought and there are a myriad of circumstances that can influence where people choose to access unscheduled healthcare for their children. The complexity of parental/family decision making which is influenced by experiences, information, knowledge, behaviour and preferences presents a challenge for policy makers and at present, there is incomplete understanding of how parents and families make decisions when accessing unscheduled healthcare.

### Factors that influence unscheduled healthcare utilization in paediatric populations

The GP acts as a gatekeeper for access to secondary healthcare services; however, in Ireland, patients access ED services either through a referral from a GP, other health professionals or through self-referral. For parents/families seeking unscheduled paediatric healthcare, there are a variety of factors that can influence whether they decide to see a GP, primary care after-hours services, privately run emergency service or the ED. For instance, the time of day and day of the week influences GP accessibility. Indeed, ED attendance by children peaks outside traditional GP working hours (e.g. ~18.00 hrs, after school, after work) ( Cecil *et al*., 2016) and children registered with more accessible GPs are less likely to visit ED out-of-hours ( Cecil *et al*., 2016). Studies revealed factors associated with non-urgent paediatric emergency visits include the need for convenient before- and after-work-hours’ service and single-parent status ( Hashikawa *et al*., 2014). In the US, a high proportion of parents report seeking medical evaluation in urgent care or emergency settings when their children’s illnesses prevent attendance at child care ( Hashikawa *et al*., 2014).

A UK study revealed that patients seeking care at the ED often doubted primary healthcare’s capacity to respond to ‘urgent’ problems and this belief results from past experiences of care-seeking ( MacKichan *et al*., 2017). In Victoria, Australia, a study of parents of children presenting to the ED with lower-urgency conditions reported a preference for ED for the care of child injuries rather than GP surgeries and most parents did not attempt to make an appointment with their GP prior to attending ED for their child’s lower-urgency injury ( Gafforini *et al.*, 2016). The socioeconomic status (SES) of patients is also known to influence their use of health services; a Turkish study found parents with higher education levels are harder to persuade that diagnostic procedures in the ED may not be necessary for some injuries ( Serinken *et al*., 2014). Patients from lower SES backgrounds typically use the ED at a greater rate than those from higher SES backgrounds ( Kangovi *et al*., 2013; Lynch *et al*., 2018; Tozer *et al*., 2015); however, there are confounding factors such as previous ill-health that can moderate this effect ( Khan *et al*., 2011). Lower caregiver health literacy (i.e., the ability to read and understand health information) ( Raynor, 2012) has also been shown to increase the likelihood of a child visiting the ED, especially for children without a chronic illness ( Morrison *et al*., 2014). A UK primary care study identified patients with English as an additional language or language/hearing difficulties could be particularly disadvantaged by primary care telephone appointment systems that are neither simple nor accessible ( MacKichan *et al*., 2017). These represent just some of the factors that influence where parents/families seek healthcare for their children; their relative importance and the different contexts in which they can occur results in a complex picture of decision making that can have a significant impact on resources in the health system. 

### Why establish factors influencing decision making for paediatric unscheduled healthcare?

Understanding the factors influencing the decision-making process, which include the preferences of parents and families, when accessing unscheduled healthcare for their child can help both clinicians and policy makers adequately respond to the healthcare needs of this population. It is important to establish the factors that influence their decision in order to inform the development of appropriate policy and ensure adequate design and resourcing in the health service. Taking the preferences of users of the health system into consideration is critical when developing and evaluating health policy, particularly in an area heavily influenced by complex patient decision-making ( Dirksen *et al*., 2013). Accessing timely healthcare in an appropriate setting affords better health outcomes for patients, and attending primary care with a regular healthcare provider or team is also thought to be of great benefit to patients. Indeed, children who experienced greater continuity of care with a single primary care provider have significantly lower ED utilisation rates and subsequent hospitalisations ( Christakis *et al*., 2001) and this is also true for children with complex medical conditions ( Arthur *et al*., 2018). As crowding in paediatric emergency departments has been associated with worse condition specific patient outcomes ( Chan *et al*., 2017), it is therefore vital to understand the determinants of the parental/family decision to self-refer to an ED rather than visit their GP and to establish the hierarchy and relative importance of these factors. The ongoing reconfiguration of paediatric healthcare in Ireland with the development of Children’s Hospital Ireland and the introduction of free GP care for children under age 6 provides an opportune time to explore paediatric healthcare. Policies that seek to expand primary care and reduce ED admissions need to be cognisant of the nuanced and complex factors that govern patients’ behaviour.

## Aim

The systematic review will seek to identify the factors that influence parents’ and families’ decision making when seeking unscheduled paediatric healthcare.

### Review question

What are the factors that influence decision-making of parents and families seeking unscheduled paediatric healthcare (general practice, out-of-hours arrangements, urgent care centre, emergency department)?

### Search term identification

A limited search of PubMed and CINAHL was carried out to identify primary keywords used in the titles and abstracts of articles that will emerge in the search engines. These were used to formulate the search terms that will be used in the systematic review.

### Timeframe

This systematic review will examine material published between 01/01/2000 and 12/03/2019.

### Inclusion criteria

- Only studies published in English will be considered for inclusion
- Empirical studies and systematic reviews
- Studies that directly sought to establish factors that influence the decision-making for the access of paediatric unscheduled healthcare.

### Exclusion criteria

- Studies that elicited factors that influence decision-making for accessing adult healthcare
- Studies related to scheduled or specialist healthcare services
- Expert opinion or editorials

### Databases

The 5 databases selected, which capture a wide range of specialities and disciplines, are as follows:

- CINAHL
- PubMed
- SCOPUS
- PsycInfo
- EconLit

### Grey literature

The search for non-peer reviewed literature will include:

- ProQuest Dissertations and Theses
- Lenus
- OpenGrey
- Google Scholar

### Key words

Keywords and Boolean operators are outlined in Table 1. 

**Table 1.** Keywords and Boolean Operators.

Child* OR paediatric OR pediatric OR Infant OR adolescent AND
Parent* preferences OR choice* OR decision making OR Family preferences OR Reasons AND
primary care OR general practice OR family physician OR emergency care OR emergency department OR out-of-hours OR Practitioner Cooperative OR after hours OR urgent care cent*

### Types of study to be included

Study designs that will be incorporated into the review include, but are not limited to:

- Population health studies
- Utility value methods
  - standard gamble interview (SGI)
  - Time trade-off (TTO)
- Stated-preference methods
  - Discrete choice experiments
  - Best-worst scaling
- Surveys and questionnaires
- Qualitative research
- Systematic reviews

### Strategy for screening

The reference list of all identified reports and articles will also be searched for additional studies. Two authors will independently screen the title and abstracts of search records retrieved against eligibility criteria. Full-text publications of all potentially relevant articles, selected by either author, will be retrieved and examined for eligibility. We will document the search strategy and study selection process using a PRISMA flow diagram ( Liberati *et al*., 2009).

### Data management

The team will use Endnote to remove duplicates and will use the review management website Covidence ^TM^ to sort exclusions and inclusions.

### Dealing with missing data

One researcher will attempt to contact study authors for unreported data or clarification of study methods using a maximum of three e-mails with 1 week between each email. If data remains unavailable, we will analyse the available data and report the potential impact of missing data in the discussion section.

### Quality appraisal

Given the heterogeneity of the study design expected to emerge in the review, the Mixed Methods Appraisal Tool (MMAT; Pluye *et al*., 2011) will be utilised to assess methodological quality of the studies included for full-text review. Papers selected for data extraction will be assessed by one reviewer, prior to inclusion in the review. A second reviewer will review 10% of the studies to check for consistency and any disagreements that arise between the reviewers will be resolved through discussion or consultation with a third reviewer.

### Data extraction

Table 2 outlines the data extraction form that will be used to extract data from the included studies. These include general information related to the study, country of origin, the aims and rationale of the research and any details on the health system in which the research took place (e.g., publicly funded, public and private etc.). Based on our initial scoping of the existing literature, it will be important to extract participant characteristics including demographic information such as socioeconomic factors, however, we anticipate that this may not be consistently recorded across the studies and we will extract any factors that are recorded (e.g., level of education, occupation etc.). With regards to the paediatric population in question, the relationship to the child (e.g., mother, father, carer), age, any disease groups or conditions will be noted and the reason for attendance at unscheduled care will be recorded. There is a broad range of potential study designs that may emerge from the searches and as a primary outcome for the review, the team will extract any factors that emerge directly from the study which have been stated to influence decision-making, behaviour or any preferences elicited from the research. One reviewer will extract the data from the included studies and 10% of these will be checked for consistency by a second reviewer. Any discrepancies will be dealt with through discussion or with a third reviewer in order to reach an agreement.

**Table 2.** Data Extraction Form.

General Information
Article Title
Authors
Country of Origin
Introduction
Aims and Rationale
Details on Health System
Research Question
Participant Details
Sample Size
Age
Gender
Relationship to child
Socioeconomic factors
Paediatric Population
Age
Specific disease group or condition (if any)
Reason for seeking unscheduled healthcare
Methods
Sampling Strategy
Study Design
Data Collection
Data Analysis
Outcomes
Factors influencing behaviour and/or decision making/Preferences elicited

### Data synthesis

A narrative approach will be used to synthesise the findings from the review.

### Registration

This systematic review has been registered with PROSPERO.

## Discussion

The present systematic review will seek to identify the factors that influence parents’ and families’ preferences and decision making when seeking unscheduled paediatric healthcare. To date, much research has focused on patients’ attitudes and decision-making in relation to specific components of unscheduled care (i.e., GP, out-of-hours or ED), whereas there is less evidence relating patient behaviour that pertains to all forms of unscheduled healthcare. The boundaries between primary and secondary care can be indistinct and ED care is often seen as a substitute for primary care, particularly in out-of-hours. By collating the factors that influence decision-making and attendance at these services, the review can inform future health policies that seek to support the provision of accessible and responsive primary care.

The review will also inform the design of a discrete choice experiment (DCE) to establish parental and family preferences for paediatric unscheduled non-specialist healthcare as part of a larger HRB-funded study investigating the patterns of attendance and decision-making around unscheduled paediatric healthcare in Ireland within the context of the introduction of free GP care for children under age 6 in Ireland. DCEs are of great value in health research as they provide an indication of how people will use the health system by presenting participants with real-world scenarios that consist of factors and attributes ( Lancsar & Louviere, 2008). Given the limited resources in the health sector, DCEs can inform policy developments that ensure resources are directed to places that matter most to patients and thus, providing a more patient-centred health system.

### Dissemination of the review

While the systematic review will be used to inform the design of a DCE to elicit preferences for paediatric healthcare, the results will also be published through the typical academic routes such as peer-reviewed journals and academic conferences. Non-academic materials will also be developed to target a broad audience, including members of the public, health service planners, and frontline clinical staff. The larger research project of which this review is part has adopted a collaborative and ongoing dissemination plan and the dissemination plan for this review will be no different. Given the importance of the topic, it is crucial that there is a constant and open discourse with all stakeholders and that the team facilitates the translation of research insights into actions, policies and prospective planning to improve healthcare outcomes for children.

## Data availability

All data underlying the results are available as part of the article and no additional source data are required.

### Reporting guidelines

Figshare: PRISMA-P checklist for “Factors that influence family and parental preferences and decision making for unscheduled paediatric healthcare: a systematic review protocol”. https://doi.org/10.6084/m9.figshare.8035154.v1 ( Nicholson, 2019).
