# Supplementary material for: Factors that influence family and parental preferences and decision making for unscheduled paediatric healthcare: a systematic review protocol
Source: HRB Open Res. 2019 Sep 30;2:11. Originally published 2019 Jun 14. [Version 2] doi: 10.12688/hrbopenres.12897.2 (PMC7016877; doi:10.12688/hrbopenres.12897.2)
Supplement: Peer review history

# Peer review: Reviewer response for version 1

**DOI:** 10.21956/hrbopenres.13968.r26752

**Published:** 2019-08-20

**Authors:** Matthew Booker

**Affiliations:** 1 School of Social and Community Medicine, University of Bristol, Bristol, UK

Thank you for the opportunity to review this systematic review protocol. This review will seek to assimilate published data on the factors that shape decision-making to access unscheduled paediatric health care. 

 The review protocol is clear. It has been prospectively registered. Inclusion criteria and eligible study designs are described. The authors will be taking a narrative approach to data synthesis, which given the heterogenous nature of study designs they wish to include, seems appropriate. 

 The review question is important, and the authors frame the need to address this question on the basis of local health policy in Ireland - in particular the development of a Children's Hospital, and free access to primary care for under 6s on the horizon. (Will the authors' specifically be seeking to identify and perform a sub-group analysis of data concerning under 6s to directly inform this progression? That wasn't quite clear). Although the research question is well-framed in the context of Irish health policy, in a couple of places it may be helpful to define terminology that may otherwise be a little too Irish-centric for broader global understanding (for example, what is Sláintecare?).

 It may be necessary to expand the Boolean terms in the search strategy. Decision-making is a tricky concept to capture adequately in Boolean terms given the way decision-shaping factors are sometimes framed in study titles and abstracts; it may benefit the review to broaden this a little and adopt an initially more inclusive approach. Likewise, it would be helpful to expand the setting search terms to include global variants such as 'Family Medicine", "Emergency Room" "Minor Injuries Unit" "Walk-in Centre" "After Hours" etc. 

 I wish the authors good luck in their review and look forward to reading the results.

I confirm that I have read this submission and believe that I have an appropriate level of expertise to confirm that it is of an acceptable scientific standard.

# Peer review: Reviewer response for version 1

**DOI:** 10.21956/hrbopenres.13968.r26670

**Published:** 2019-07-25

**Authors:** Elizabeth Cecil

**Affiliations:** 1 Department of Primary Care and Public Health, Imperial College London, London, UK

This is a well written protocol for an important piece of work. The protocol clearly sets out the rational for the systematic review and clearly defines its objectives.

 The protocol is registered with PROSPERO. PRISMA-P guidelines have been followed (a completed checklist is available). The protocol is very comprehensive but the few comments I have are listed below:

**Are sufficient details of the methods and analysis provided to allow replication by others?**

 Although the methods are clearly set out with all necessary details, I do think search strategy keywords need to be developed further. There are five elements to the research question.

1. Child AND
2. Parent AND
3. Decision making AND
4. Unscheduled AND
5. Health services

The synonyms for these need to be more extensive than they currently are. For example the term family practice is not included. Terms such as health or care seeking have also not been included.

 There are two sections headed data extraction, these should be combined.

**Other points**

 Slaintecare is mentioned in the abstract but is not explained or referred to later in the protocol. More details will need to be added for non-Irish readers.

I confirm that I have read this submission and believe that I have an appropriate level of expertise to confirm that it is of an acceptable scientific standard.
